# Niche Differentiation of Arsenic-Transforming Microbial Groups in the Rice Rhizosphere Compartments as Impacted by Water Management and Soil-Arsenic Concentrations

**DOI:** 10.3389/fmicb.2021.736751

**Published:** 2021-11-05

**Authors:** Anil C. Somenahally, Richard H. Loeppert, Jizhong Zhou, Terry J. Gentry

**Affiliations:** ^1^Texas A&M AgriLife Research, Overton, TX, United States; ^2^Department of Soil and Crop Sciences, Texas A&M University, College Station, TX, United States; ^3^Institute for Environmental Genomics, University of Oklahoma, Norman, OK, United States

**Keywords:** arsenic methylating bacteria, arsenate reducing bacteria, arsenic speciation, taxonomic classification, functional redundancy

## Abstract

Arsenic (As) bioavailability in the rice rhizosphere is influenced by many microbial interactions, particularly by metal-transforming functional groups at the root-soil interface. This study was conducted to examine As-transforming microbes and As-speciation in the rice rhizosphere compartments, in response to two different water management practices (continuous and intermittently flooded), established on fields with high to low soil-As concentration. Microbial functional gene composition in the rhizosphere and root-plaque compartments were characterized using the GeoChip 4.0 microarray. Arsenic speciation and concentrations were analyzed in the rhizosphere soil, root-plaque, pore water, and grain samples. Results confirmed several As-biotransformation processes in the rice rhizosphere compartments, and distinct assemblage of As-reducing and methylating bacteria was observed between the root-plaque and rhizosphere. Results confirmed higher potential for microbial As-reduction and As-methylation in continuously flooded, long term As-contaminated fields, which accumulated highest concentrations of As^III^ and methyl-As concentrations in pore water and rice grains. Water management treatment significantly altered As-speciation in the rhizosphere, and intermittent flooding reduced methyl-As and As^III^ concentrations in the pore water, root-plaque and rice grain. Ordination and taxonomic analysis of detected gene-probes indicated that root-plaque and rhizosphere assembled significantly different microbial functional groups demonstrating niche separation. Taxonomic non-redundancy was evident, suggesting that As-reduction, -oxidation and -methylation processes were performed by different microbial functional groups. It was also evident that As transformation was coupled to different biogeochemical cycling processes (nutrient assimilation, carbon metabolism etc.) in the compartments and between treatments, revealing functional non-redundancy of rice-rhizosphere microbiome in response to local biogeochemical conditions and As contamination. This study provided novel insights on As-biotransformation processes and their implications on As-chemistry at the root-soil interface and their responses to water management, which could be applied for mitigating As-bioavailability and accumulation in rice grains.

## Introduction

Microbial interactions in the rhizosphere are major drivers of biogeochemical cycling processes and can significantly impact the biogeochemistry of metals including the bioavailability of toxic metalloids such as arsenic (As), which is linked to human cancer when exposed to high concentrations (Jomova et al., 2011). Microbial interactions that can affect the As biogeochemistry in the rice rhizosphere are particularly important as many reports implicate As-contaminated rice grain consumption as a major As-exposure route for millions of people (Mondal and Polya, 2008; Ohno et al., 2009). One major source of As to rice fields in Southeast Asia is the natural weathering of geological minerals containing As and subsequent transport to aquifers and surface water sources used for irrigation of rice paddies (Seddique et al., 2008; Winkel et al., 2011). Many anthropogenic sources are also responsible and one such example is the historical application of As pesticides to the cotton fields in Southern United States, some of which are currently used for rice production (Bednar et al., 2002; Williams et al., 2007). Rice crop is mostly cultivated under continuously flooded field conditions (more than 75% of the total acreage) (Roger et al., 1993). Continuous submergence can result in higher As uptake by rice plants in fields with moderate to high soil As-concentrations (Xu et al., 2008; Pillai et al., 2010), as prolonged anaerobic conditions can increase As solubility (Masscheleyn et al., 1991). Alternatively, growing rice using intermittent flooding proved to be an effective strategy to mitigate As-uptake by rice (Somenahally et al., 2011b; Linquist et al., 2015; Wang et al., 2019). It was noted that concentration of highly bioavailable arenite (As^III^) and dimethylarsinic acid (DMAs^V^) forms decreased under intermittently flooded conditions, but at variable range between the As contaminated and control sites (Somenahally et al., 2011b), which was alluded to changes in microbial mediated responses in the rhizosphere (Wang et al., 2019).

Microbial transformations can increase As-solubility, as many soil microbes can transform As chemistry for resistance and detoxification purposes (Oremland and Stolz, 2003). Arsenic bioavailability is particularly impacted by microbial interactions linked to As speciation, as some As species such as arsenite (As^III^) and dimethylarsinic acid (DMAs^V^) are less strongly adsorbed by soil minerals and are more bio-available compared to arsenate (As^V^) (Jia et al., 2014). Several reports implicated microbial interactions in the rice rhizosphere as major drivers of As-transformation (Jia et al., 2014; Zhang et al., 2015) and bioavailability of As to plants (Kumarathilaka et al., 2018). For example, arsenate (As^V^) reduction *(arsC* gene) by As^V^-reducing bacteria (ARB) and membrane bound arsenite (As^III^) efflux carriers (*arsAB* genes) can increase As availability to rice plant (Hu et al., 2015). Similarly, As methylation (*arsM* gene) by As-methylating bacteria (AMB) are mostly responsible for increasing methyl-As (mAs) concentrations in rice grains (Lomax et al., 2012). Additionally, As solubility is also coupled to other metal biotransformation processes, particularly iron (Fe) (Lafferty and Loeppert, 2005) and sulfate (SO_4_) reduction (Battaglia-Brunet et al., 2012), and sulfide-oxidation (Hoeft et al., 2004). Iron-reducing bacteria (IRB) can potentially release As complexed with Fe-oxides through reductive dissolution (Neubauer et al., 2007; Dai et al., 2020) and increase As bioavailability in the rice rhizosphere (Liu et al., 2018). Whereas SRBs could promote As-precipitation (Serrano and Leiva, 2017) and reduce bioavailability (Jia et al., 2015).

Alternative water management practices to grow rice instead of continuous flooding, were shown to significantly alter soil microbial community in the rice rhizosphere (Somenahally et al., 2011a; Wang et al., 2019). Particularly the rhizosphere functional groups responded differently to water management (Das et al., 2016), and many anaerobic functional groups such as IRBs and ARBs were diminished under intermittent flooding compared to continuously flooded fields (Zecchin et al., 2017b). Similarly, SRB abundance in the rice rhizosphere was altered by water management practices, with potential implications for As-chemistry and bioavailability (Jia et al., 2015). Another study noted that anaerobic and aerobic As-methylators responded differently to water management and altered methyl-As production in the rhizosphere (Wang et al., 2019), and increased mAs production under continuous flooding (Li et al., 2009; Moreno-Jiménez et al., 2014). However, it is not clear if intermittent flooding can significantly diminish these key functional groups in the long-term As contaminated fields, which may have assembled microbiome enriched by As-resistant microbes (Hu et al., 2019). It was clear that long-term As contamination can significantly alter rice rhizosphere microbial community (Somenahally et al., 2011a) and promote As-resistant functional groups (Hu et al., 2019). One study noted that As contamination significantly altered microbial community (Xiong et al., 2012), and As resistant microbes such as ARBs may increase in contaminated soils (Das et al., 2019). Exposure to higher As concentrations may also increase functional groups interlinked to As-resistance, similar to the As-S transformation processes interlinked in SRBs (Serrano and Leiva, 2017). Particularly, functional groups inter-linked to As-reduction and methylation are not well characterized in rice fields with a history of As-pesticide contamination, which tend to accumulate higher ratio of mAs:As^III^ in the rice grains (Pillai et al., 2010; Somenahally et al., 2011b).

Microbial functional groups in root-plaque compartment, which can accumulate significantly higher concentration of As, are not clearly understood. Root-plaque refers to Fe-oxides accumulation on root surface because of oxidation of Fe^II^ to Fe^III^ and precipitation of dissolved Fe^II^ diffusing on the roots (Taylor et al., 1984). Major mineral comprising these root-plaques is crystalline ferrihydrate (Bacha and Hossner, 1977; Hossain et al., 2009), that can effectively complex with As^V^ and subsequently accumulate higher concentrations (Liu et al., 2004). Microbial interactions at root-plaque, including As- and Fe-reducing bacteria could potentially disrupt these Fe-As phases and release complexed-As (Chen et al., 2005; Hu et al., 2015). Water management is anticipated to impact their assemblage in root-plaque as redox gradient and biogeochemical conditions are major drivers of microbial assembly in a wetland plant rhizosphere (Neubauer et al., 2007; Dai et al., 2020). However, none of the studies looked at niche separation of microbial assembly, particularly the As-transforming functional genes in the rice rhizosphere and root-plaque compartments in long-term As-contaminated fields. Aim of this study was to outline key microbial functional group responses in the rhizosphere compartments along with As-speciation changes and uptake by the rice plant in response to water management and long-term As contamination. We hypothesized that rhizosphere compartments assemble distinct As-transforming functional groups and respond differently to water management practices impacting As-speciation in the rhizosphere. Particularly, ARBs and AMBs were anticipated to significantly increase in continuously flooded As-contaminated treatments and subsequently contribute to increased mAs accumulation in the rice grain. A field experiment was conducted to compare continuous and intermittent flooding practices imposed on fields with high to low soil-As concentrations. Major objective was to determine As-, Fe- and S-transforming functional groups within the rhizosphere compartments. Additionally, As-speciation and quantitation were performed to understand As bioavailability under the treatment effects and link with the functional gene responses.

## Materials and Methods

### Field Experimental Details

This field experiment was conducted at the US Department of Agriculture Dale Bumpers National Rice Research Center near Stuttgart, AR (34.471.217, -91.422.350). The experimental plots were arranged in a split-split plot design of three replicates, with soil-As amendment as the main plot, and water management (flooding) as the sub-plot. Soil-As amendment included two treatments, As amended and unamended control. The As-amended plots received an As-based pesticide commonly known as monosodium methane-arsonate (MSMA) in alternate years for more than past 25 years (Yan et al., 2005). These applications were practiced for simulating historical As-pesticide applications in cotton fields in the region and screen rice varieties resistant to higher soil-As. Each application of MSMA was in solution, applied to the soil surface before planting, at the rate of 6.7 kg/ha (equivalent to 3.1 kg ha^–1^ of As). These fields will be referred to as “As-amended.” An adjacent area, referred to as “control,” had not been exposed to any As-containing products for at least the last 30 years. The irrigation water practices included intermittent and continuous flooding on both the MSMA and control plots. Under intermittent flooding, the plots were flooded and allowed to dry until surface cracks appeared, before re-flooding. For the continuously flooded treatment, water was consistently maintained at least 10 cm in depth during the entire rice growing season until a week before harvesting. The experimental treatments used in this study were (i) continuously flooded-As amended (CFA), (ii) continuously flooded-control (As unamended) (CFC), and (iii) intermittently flooded-control (no MSMA) (IFC). The soil type in the experimental plots was a fine, montmorillonite, thermic Typic Albaqualf with soil texture of silt loam to loam. Additional details on soil properties are presented in Supplementary Table 1. More details on management practices used for rice cultivation at these sites can be found in Yan et al. (2005).

### Sampling Protocols for Rhizosphere Compartments, Pore Water, and Rice Grains

Approximately 3 months after the first flood, at about 6-leaf stage (120 days after planting), the rhizosphere and the root-plaque samples were collected from each treatment plot. At the sampling time, the CF plots had been continuously flooded for approximately 12 weeks with standing water, while the IF plots had been going through wet-dry cycles. Four to five rice plants per plot were collected, along with the adhering bulk soil. The plants were shaken to remove loose soil, and the remaining few millimeters of rhizosphere soil left around the roots was collected manually into sample tubes and transferred to ice immediately. The roots were then thoroughly washed with sterile deionized water to remove any remaining soil. Roots with Fe-oxide plaque affixed were collected as the root-plaque samples. The samples were split into subsamples for subsequent chemical and microbial DNA extractions. Samples for chemical analysis were transported on ice and immediately processed for As analysis. Rhizosphere and root-plaque samples for microbial DNA extractions were frozen over dry ice in the field and were subsequently stored at −80°C. Rhizosphere soil samples were air dried, ground to < 2 mm size and stored at room temperature for chemical analysis. The root-plaque subsamples for chemical analysis were immediately extracted for As forms, total As and Fe, and the extracted solutions were stored at 4°C until further analysis.

For pore water collection, we used soil core samples from the rooting zone, collected at the same time, using an 8.5-cm diameter cover with plastic liners for sample preservation. The plastic liners with the soil samples were capped with polypropylene end caps, and the samples were stored on ice during transport to the laboratory. The core samples were then vacuum filtered through 0.2μm pore size mixed cellulose-ester filters at a negative pressure of 138 kPa for 20 min to extract pore-water and then acidified to pH 3 with HNO_3_ and stored for subsequent As-analysis. Rice grain samples were collected at the time of harvest. Redox potential in the root zone and bulk soil was measured at three time points during the growing season, and at the sampling time using a platinum electrode using methods similar to described by Somenahally et al. (2011a). Additional details on sampling methods can be found in Supplementary Information.

### Arsenic Speciation and Analysis

The As forms (As-species) in the rhizosphere soil and the root- plaque samples were determined following a sequential extraction with 0.4 M H_3_PO_4_ and 0.4 M NaOH. As-forms detected in different samples included arsenate (As^V^), arsenite (As^III^), monomethylarsonic acid (MMAs^V^), and dimethylarsinic acid (DMAs^V^). We combined both forms for organic-As (MMAs^V^ and DMAs^V^) to present as total methyl-As (mAs) concentrations. Methods followed for As forms and the total-As extraction procedure from rhizosphere soil, root-plaque, pore water and grain samples and As:Fe molar ratios estimation in root-plaque samples were similar to methods described by Somenahally et al. (2011b). Concentrations of the As-forms (As^V^, As^VII^, and m-As) were determined using a high performance liquid chromatography (HPLC) system (Perkin-Elmer, Waltham, MA) attached inline to a ELANDRCII inductively coupled-plasma mass-spectrometer (ICP-MS) (Perkin-Elmer). Additional details are provided in Supplementary Information.

### GeoChip Microarray Hybridization and Data Analysis

The microbial community DNA was extracted from the frozen rhizosphere and the root-plaque samples using MO BIO Power Max DNA extraction kits (Qiagen Inc.). We used a modified version of the manufacturer’s protocol, which included a lysozyme pre-incubation step, in order to enhance gram-positive bacterial DNA yield (Somenahally et al., 2011a). Approximately 10 g of rhizosphere soil or 5 g root samples (with plaque) were treated with lysozyme solution (10 mg per sample final concentration) and incubated in a water bath for 1 h at 37.5°C with occasional shaking, after which the manufacturer’s protocol was resumed from the bead-beating step. The resulting DNA samples were concentrated by ethanol precipitation, purified using Illustra MicroSpin^TM^ S-400 columns (GE Healthcare Biosciences, Pittsburgh, PA, United States), and stored at −20°C.

Rhizosphere and root-plaque DNA samples were submitted to Institute for Environmental Genomics at Oklahoma University, Norman, OK for GeoChip analysis. Three biological replicate DNA samples from treatments CFA, CFC, and IFC were analyzed with GeoChip 4.0, which had approximately around 110,000 probes. Further details on hybridization parameters can be found in Tu et al. (2014). Microarray hybridization array data was processed and normalized by following the data analysis pipeline detailed in Tu et al. (2014). We further processed the data by replacing all the biological replicates values by a zero value if any of the three replicates had no signal intensity. Further normalization of the data was performed by transforming absolute detection to relative abundance by estimating ratio of each probe to all genes detected by GeoChip for that specific sample. Relative signal intensity ratios were used for all comparative analysis and multivariate statistical analysis. We used specific gene probes for quantifying relative abundance and taxonomic assessment of As, S and Fe transforming functional groups. For As-transforming functional groups, gene probes detected by Geochip included aoxB, arsA, arsB, arsC, and arsM. We combined *arsA, arsB*, and *arsC* gene probes for arsenate-reducing bacteria (ARB). For sulfate reducing bacteria (SRB), gene probes included *aprA, APS_aprA, APS_aprB, dsrA*, and *dsrB*. For sulfur oxidizing bacteria (SOB) we used *sox* gene probes. For potential iron reducing bacteria (pIRB) we used cytochrome gene probes from known IRBs. Other gene probes used in this study for estimating gene categories represented within ARBs and SRB are listed in Supplementary Table 2. The datasets presented in this study can be found in NCBI repository under the accession GSE179671.

### Statistical Analysis

Principal component analysis (PCA) was performed using the PAST software (Hammer et al., 2001). The PCA routine in PAST finds the eigenvalues and eigenvectors of the variance-covariance matrix or the correlation matrix. We used the variance-covariance matrix for the gene-probe relative abundance data. The Biplot option was used for projecting the predominant species (gene-probes) constraining the principal components. A one-way non-parametric multivariate analysis of variance (PERMANOVA) was used to test the significant differences between the experimental treatments for relative abundance of functional groups, based on Bray-Curtis similarity index. The canonical correspondence analysis (CCA) for the relative abundance of arsenate-reducing bacteria (ARBs) and sulfate reducing bacteria (SRBs) were compared with their functional associations (other gene probes detected in these organisms). The gene categories and their relative abundance were estimated for each treatment sample, which was then used as variables (priori) within CCA and were plotted as biplots. Hierarchical clustering analysis and heat maps were created for the relative signal intensities for gene probes in GeoChip using Gplots package within R software. The individual probe data were then grouped by taxonomic phyla for graphing.

## Results

### Arsenic Concentrations in the Rhizosphere Compartments and Rice Grain

One set of rice field sites (CFA treatment) used for this study received As-pesticide (MSMA) application to soil for more than 25 years. As a result, total-As concentrations in the rhizosphere soil were significantly higher in CFA (continuously flooded that received MSMA) compared to control field sites (CFC and IFC), which did not receive any MSMA application for the last 25 years (Table 1). Average rhizosphere soil-As concentration was significantly higher (*p* < 0.05), at around 20 mg kg^–1^ in CFA plots, compared to around 6 mg kg^–1^ in control plots (CFC and IFC). The root-plaque compartment accumulated almost 10 times higher-As concentrations (*p* < 0.05) compared to the adjacent rhizosphere soil and highest concentrations were detected in CFA plots (301 mg kg^–1^) compared to the control plots (CFC and IFC). Water management significantly (*p* < 0.05) altered the redox potential in the rhizosphere, as intermittent flooding (IF fields) created a more aerobic environment and recorded substantially higher redox potential compared to CF field sites (Table 1). As a result, higher As:Fe ratios were detected in CFA compared to other treatments (Table 1).

**TABLE 1:** Total As-concentrations and redox potential in the rhizosphere and root-plaque samples under the experimental treatments.

Treatments	Rhizosphere	Root-plaque^≠^		Redox potential (mv)	
	Total As (mg kg^–1^)	Total As (mg kg^–1^)	As:Fe (%)	Rhizosphere	Bulk soil
CFA^£^	^a¥^20.1 (± 1.4)	^a^301 (± 38)	^a^0.37 (± 0.08)	^b^ + 11 (± 4)	^b^−56 (± 4)
CFC	^b^6.7 (± 0.32)	^b^154 (± 14)	^a^0.28 (± 0.03)	^c^−13 (± 3)	^c^−77 (± 5)
IFC	^b^5.5 (± 0.41)	^c^73 (± 11)	^b^0.20 (± 0.03)	^a^ + 39 (± 6)	^a^ + 23 (± 2)

*^≠^Root-plaque As and Fe concentrations are based on root dry mass.*

*^£^Treatments: CFA = continuously flooded on arsenic (MSMA) amended plots, CFC = continuously flooded on no-As amended control plots and IFC = intermittent flooding and on no-As amended control plots.*

*^¥^Letters in superscript indicate LSD mean difference comparisons for the particular As-species between the three different experimental treatments. Different letters indicate significant mean difference at p < 0.05. This analysis was performed only when As-species was detected in all experimental samples. Values in parenthesis are standard error of mean.*

In the rhizosphere soil, As^V^ was detected at highest concentrations (10.3 mg kg^–1^) and were significantly (*p* < 0.05) higher in CFA treatment compared to other two treatments (Table 2). Minimal concentrations of As^III^ and mAs were detected only in CFA plots. Root-plaque compared to the rhizosphere, accumulated substantially higher As-concentrations (Table 2). Considerable amount of total-As accumulation in the root-plaque was detected in the form of As^V^ (around 80%). Concentrations were significantly (*p* < 0.05) higher in the CFA treatment (228.4 mg/g), followed by CFC treatment (118.8 mg kg^–1^), which were significantly (*p* < 0.05) higher than IFC treatment (56.6 mg kg^–1^). Additionally, As^III^ was detected at around 16% and mAs at around 4% of the total As concentrations. Treatment effects were similar as As^V^, and As^III^ concentrations were highest in CFA (46 mg kg^–1^) followed by CFC (22.9 mg kg^–1^) and IFC (13.2 mg kg^–1^). Methyl-As was detected only in the CFA treatment at 12.5 mg kg^–1^. In pore water samples obtained from the rhizosphere, the predominant As-form was As^III^ (around 80%). Pore water As^III^ concentrations were significantly (*p* < 0.05) higher in the CFA treatment (19.4 μg L^–1^), followed by CFC treatment (8.6 μg L^–1^), which were significantly higher than IFC treatment (2.1 μg L^–1^). Methyl-As was detected in the pore water of CFA treatment at 3.9 μg L^–1^ and some negligible amount in CFC. In rice grains from CFA treatment, the predominant As-form was mAs (around 83%), and in other treatments both mAs and As^III^ were detected at comparable percentage (Table 2). Concentrations of mAs were significantly higher in the CFA treatment (723.9 μg kg^–1^), followed by CFC treatment (158 μg kg^–1^), which were significantly higher than IFC treatment (88.5 μg kg^–1^). Concentrations of As^III^ were significantly higher in the CFA treatment (148.3 μg kg^–1^), followed by CFC treatment (138.1 μg kg^–1^) and IFC treatment (123.2 μg kg^–1^). There was no significant difference between CFC and IFC treatments. As a result, mAs:As^III^ in grain samples were significantly higher in continuously flooded treatments.

**TABLE 2:** Arsenic species concentrations in the rhizosphere, root-plaque, pore water, and grain samples under different soil-As concentrations and flooding treatments.

Treatments	Soil (mg kg^–1^)			Root-plaque (mg kg^–1^)			Pore water (μ g L^–1^)			Grain (μ g kg^–1^)		
	As^V^	As^III^	mAs^≠^	As^V^	As^III^	mAs	As^V^	As^III^	mAs	As^V^	As^III^	mAs
CFA	^a¥^10.3 (± 2.1)	1.4 (± 0.2)	0.9 (± 0.1)	^a^228.4 (± 24.6)	^a^46.0 (± 3.12)	12.5 (± 1.2)	1.2 (± 0.1)	^a^19.4^a^ (± 2.5)	3.9 (± 0.12)	<0.01	^a^148.3 (± 17)	^a^723.9 (± 31)
CFC	^b^3.6 (± 0.6)	<0.01	<0.01	^b^118.8 (± 16.2)	^b^22.9 (± 3.5)	<0.01	0.3 (± 0.01)	^b^8.6 (± 0.57)	1.8 (± 0.10)	<0.01	^b^138.1 (± 21)	^b^158.0 (± 11)
IFC	^b^3.9 (± 1.1)	<0.01	<0.01	^c^56.6 (± 6.8)	^c^13.2^c^ (± 0.9)	<0.01	<0.01	^c^2.1 (± 0.31)	<0.01	<0.01	^b^123.2 (± 13)	^c^88.5 (± 18)

*^≠^mAs = total methylated As-species, for sum of MMA and DMA.*

*^¥^Letters in superscript indicate LSD mean difference comparisons for the particular As-species between the three different experimental treatments. Different letters indicate significant mean difference at p < 0.05. This analysis was performed only when As-species was detected in all experimental samples.*

*Values in parenthesis are standard error of mean.*

### As-Transforming Functional Groups in the Rhizosphere Compartments

**FIGURE 1:**
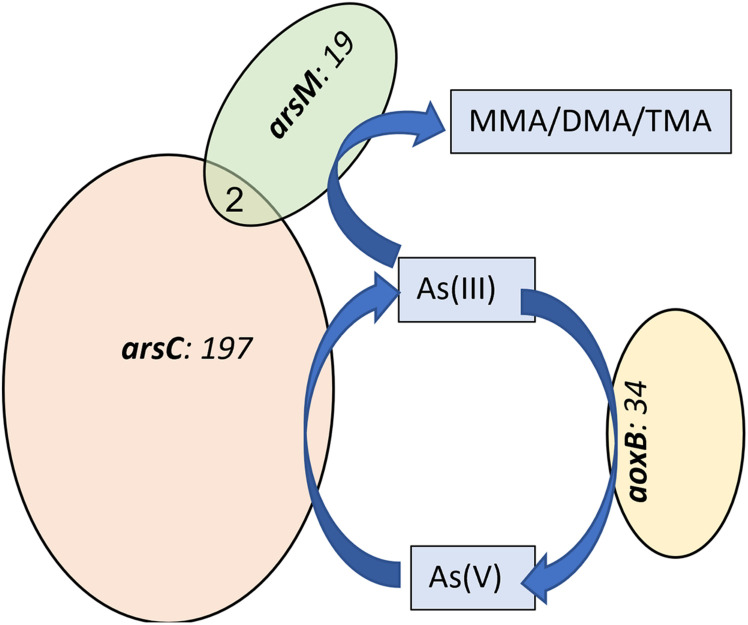
Venn diagram showing the number of unique and shared functional gene probes containing different arsenic transforming gene probes from all treatments. Numbers within the Venn diagrams represents total number of positive gene probes detected.

Among several functional genes detected by GeoChip 4, our focus for this study was on As-, S-, and Fe-transforming genes, as these functional groups largely influence As chemistry and bioavailability in the rice rhizosphere. ARB, which carry As^V^-reductase (*arsC*) and As^III^-efflux genes (*arsA/B*), were the most abundant functional group detected among all the As- transforming gene probes detected in this study (Figure 1). Other As related genes detected were arsenite oxidizing bacteria (AOB), which carry *aoxB* gene and As-methylating bacteria (AMB) which carry *arsM* gene, part of As-methylation pathway, presumed to be one of the detoxification mechanisms (Das et al., 2017). The *arsC* gene was detected in 197 prokaryotic group (based on unique gene probes in Geochip), and *aoxB* in 34 and *arsM* in 19 unique gene probes (organisms) (Figure 1). *arsA/B* were detected in less than 10 unique probes. Only two organisms were positive for multiple As-functional gene probes (*arsC* and *arsM*). These results confirm taxonomic non-redundancy implying that As-reduction, -methylation, and -oxidation processes were carried out by different group of microorganisms in the rice rhizosphere microbiome.

**FIGURE 2:**
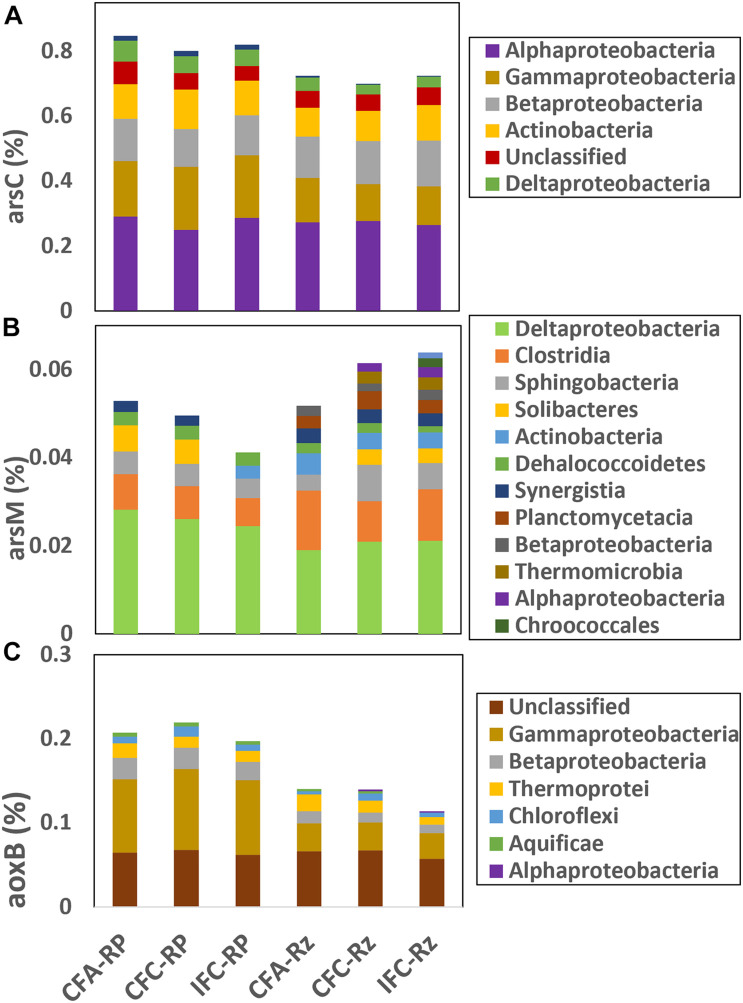
Relative abundance of major taxonomic classes represented within different arsenic cycling gene probes **(A)** arsC, **(B)** arsM and **(c)** aoxB in the rhizosphere compartments under different soil As and water management treatments. CFA, continuously flooded-arsenic; CFC, continuously flooded-control; IFC, intermittently flooded control. Suffix RP and Rz in group labels represent the root-plaque and the rhizosphere samples, respectively.

Taxonomic affiliation for positive gene probes within ARBs, AOBs and AMBs were compiled (Figure 2). Higher abundance of *arsC* gene probes were noted within *Alphaproteobacteria* followed by *Gammaproteobacteria, Betaproteobacteria*, and *Actinobacteria* (Figure 2). Whereas, most abundant *arsM* gene probes were within *Deltaproteobacteria* and *Clostridia*, which indicate that As methylation in the rice rhizosphere compartments was likely confined to mostly anaerobic bacteria. The most abundant *aoxB* gene probes were either unclassified or were within *Gammaproteobacteria*. Overall abundance of *arsC* gene in the root-plaque compartment were higher compared to the rhizosphere, however, not many differences were noted between As- and water-treatments (Figure 2A). The *arsC* represented in *Gammaproteobacteria* were detected at higher abundance in the root-plaque samples compared to the rhizosphere, but there was no difference among other taxonomic groups. Abundance of *arsM* gene was higher in the rhizosphere compartment compared to the root-plaque (Figure 2B). The *arsM* represented in *Deltaproteobacteria* were detected at higher abundance in the root-plaque compared to the rhizosphere, whereas *Clostridia* were higher in the rhizosphere. The *arsM* abundances were not significantly different between either As- or water-treatments. Hierarchical clustering of *arsM* gene signal intensity indicated that As-methylators clustered mostly by compartments, and then by treatments within the compartments (Supplementary Figure 1). The probe representing *Desulfohalobium retbaense*, a sulfate reducing bacterium was the major As-methylator target detected in most samples. Other major As-methylators were similar to *Salinibacter ruber, Desulfovibrio desulfuricans, Pelotomaculum thermopropionicum*, and *Desulfotomaculum acetoxidans*. Hierarchical clustering of *arsC* also demonstrated similar response to As and water treatments (Supplementary Figure 2). Probes representing *Rhodococcus erythropolis, Nitrobacter winogradskyi*, and *Desulfobacterium autotrophicum* were the most abundant ARBs detected in all samples, whereas one similar to *Burkholderia multivorans* and *Maricaulis maris* were predominant in the rhizosphere and *Enterobacter sakazakii* in the root-plaque. Abundance of *aoxB* was higher in the root-plaque compartment compared to the rhizosphere (Figure 2C). The *aoxB* represented within *Gammaproteobacteria* and *Betaproteobacteria* were significantly higher in the root-plaque compared to rhizosphere, but there were not many differences between the As and water treatments.

**FIGURE 3:**
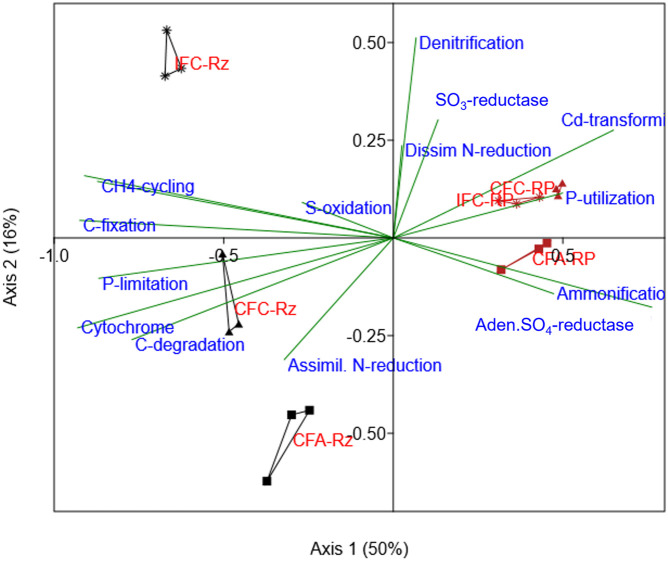
Canonical correspondence analysis for ARB (*arsC* gene) abundances compared with other functional associations (projected as bi-plot vectors) in their genomes (other gene category probes detected in these organisms). The *arsC* positive organisms from each sample were selected from other gene categories and their abundance was summed by samples. This data was used as variables for correspondence analysis and then were plotted according to their correlations with *arsC* gene probes. Suffix RP and Rz in group labels represent the root-plaque and the rhizosphere samples, respectively. CFA, continuously flooded-arsenic (squares); CFC, continuously flooded-control (triangles); IFC, intermittently flooded control (stars).

The PCA analysis of As-gene probes indicated a distinct separation by rhizosphere compartments (rhizosphere vs. the root-plaque) (Supplementary Figure 3). Since almost 81% of variance was reported within the first two components, it can be assumed that As-transforming functional groups were more responsive to rhizosphere compartmentalization. Several microbial groups were predominant in root plaque compartment such as organisms similar to *Enterobacter sakazakii*, *Desulfobacterium autotrophicum*, *Nitrobacter winogradskyi*, and *Vibri*o spp. We further explored through CCA analysis ARBs assemblage in the treatments and relationship to other genomic functions detected in these microorganisms associated to positive gene probes. The analysis indicated that other major genomic functions within the ARBs were distinctly aligned between the compartments (Figure 3). The ARBs within the rhizosphere compartment mostly associated with anaerobic processes and carbon metabolism, such as carbon degradation pathway, acetogenesis, methanogenesis, carbon fixation, and cytochrome dependent pathways (Figure 3). Whereas in the root-plaque, ARBs associated with ammonification and phosphorus utilization among other functions. It was also interesting to note that ARBs in the rhizosphere appeared to be more sensitive to water and As-treatments compared to‘the ARBs in the root-plaque. These results confirm distinctive functional capabilities of ARBs in the rhizosphere compared to the root-plaque compartment. A separate PCA analysis has indicated that several ARBs in the rhizosphere and root-plaque compartments were responsive to water management and soil-As concentrations (Supplementary Figure 4). PERMANOVA analysis also confirmed that ARBs in both compartments were significantly influenced by soil-As and water treatments (Table 3). Several ARBs probes were predominant in As-amended treatments (CFA), such as *Enterobacter sakazakii* in the rhizosphere, and *Desulfobacterium autotrophicum*, *Deinococcus geothermalis*, and *Rhodococcus erythropolis* in the root-plaque. *Nitrobacter winogradskyi* and *Frankia* sp. probes associated with IFC treatments in the root plaque. These results further confirm that several ARBs were responsive to redox changes by water management, which may have altered As-reduction potential in the rhizosphere.

**TABLE 3:** PERMANOVA *p*-values for experimental variables (arsenic and water treatments) on the gene-probe relative intensities in the rice rhizosphere and the root-plaque compartments.

	Rhizosphere		Root-plaque	
Functional groups	Arsenic (As)	Water (W)	Arsenic (As)	Water (W)
ARB	0.01	0.02	0.01	0.07
IRB	0.05	0.06	0.01	0.05
SRB	0.05	0.01	0.01	0.04
SOB	0.03	0.05	0.04	0.01

*ARB, Arsenate-reducing bacteria; IRB, iron reducing bacteria; SRB, sulfate/sulfite reducing bacteria; SOB, sulfur oxidizing bacteria.*

### Iron and Sulfur Transforming Functional Groups in the Rhizosphere Compartments

**FIGURE 4:**
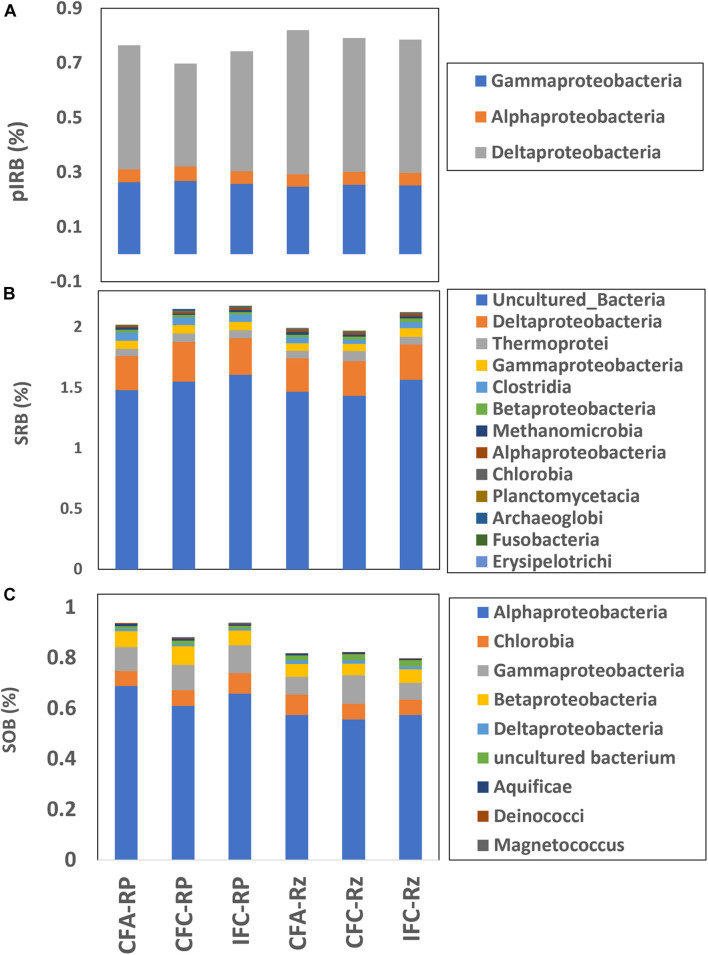
Relative abundance of major taxonomic classes represented within **(A)** cytochrome gene probes, **(B)** SRB (dsrA/B, APS_AprA/B gene probes), and **(C)** SOB (SOX gene probes) in the rhizosphere compartments under different soil As and water management treatments. CFA, continuously flooded-arsenic; CFC, continuously flooded-control; IFC, intermittently flooded control. Suffix RP and Rz in group labels represent the root-plaque and the rhizosphere samples, respectively.

Biotic Fe-reduction can disrupt Fe-oxide phases and release complexed-As, particularly in the root-plaque where As:Fe ratios are significantly higher. A PERMANOVA analysis of potential iron-reducing bacteria (pIRB) gene probe abundance indicated a significantly different (*p* < 0.1) response to water and As treatments in both the rhizosphere and root-plaque compartments (Table 3). Most pIRBs detected in these systems were *Deltaprotebacteria*, followed by a few in *Gammaproteobacteria* and some *Alphaproteobacteria* (Figure 4A). The PCA analysis of pIRB probe abundance revealed a significant difference between the rhizosphere compartments (Supplementary Figure 5). Major pIRB probes driving the variation between the groups included *Rhodobacter sphaeroides, Geobacter* sp. M21, *Anaeromyxobacter dehalogenans*, and *Thermoplasma acidophilum.*

**FIGURE 5:**
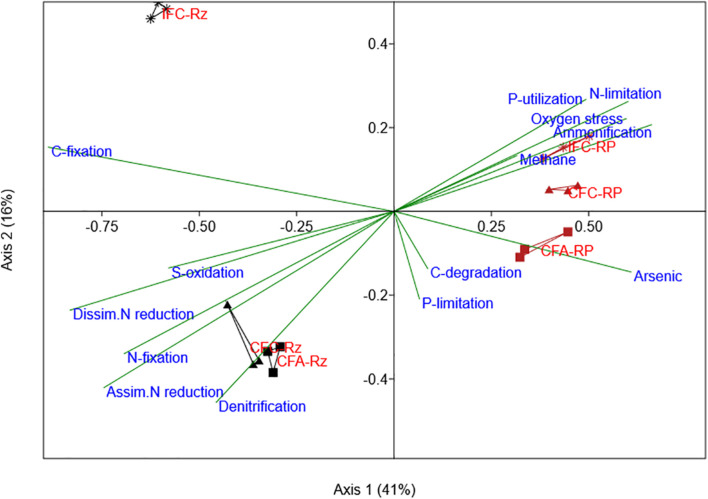
Canonical correspondence analysis for SRB (dsrA/B, APS_AprA/B gene probes) abundances compared with other functional associations in their genomes (other gene category probes detected in these organisms). The SRB gene probes positive organisms from each sample were selected from other gene categories and their abundance was summed by samples. This data was used as variables for correspondence analysis and then were plotted according to their correlations with *SRB* gene probes.

Similarly, a PERMANOVA analysis of SRB gene probe abundance indicated a significant difference (*p* < 0.05) between the water and As-treatments (Table 3). The relative abundance of SRB functional genes was slightly higher in the root-plaque samples compared to the rhizosphere soil (Figure 4B). Most SRBs were classified as uncultured or unclassified gene probes of bacteria, followed by *Deltaproteobacteria*. The PCA analysis indicated a distinct separation by rhizosphere compartments (Supplementary Figure 6). Principal SRB probes leading the variability between the groups included several uncultured isolates, a *Desulforudis audaxviator*, a *Desulfofustis glycolicus*, and a *Pyrobaculum calidifontis*. According to CCA analysis, distinctive assembly of SRBs within the compartments showed further functional non-redundancy (Figure 5). Gene probes within carbon fixation pathway, assimilatory N reduction, denitrification, nitrogen fixation were major functional associations for SRBs in the rhizosphere compartment. Whereas SRBs in the root-plaque were associated with oxygen stress, nitrogen limitation, phosphorus utilization, and As-resistance genes as other major selective functions. A clear distinction was also observed in response to water treatments within the compartments, particularly in the rhizosphere compartment. It was evident that saturated rhizosphere compartment (IFC-Rz) assembled a functionally distinct SRBs comparted to continuously flooded plots (CFC-Rz and CFA-Rz). These results confirmed functional non-redundancy within the SRBs within the compartments and in response to As and water treatments.

The relative abundance of SOBs (SOX gene probes) did not change significantly between the treatments or compartments (Figure 4C). Most of the SOX gene probes detected were either *Alphaproteobacteria*, *Chlorobia*, *Gammaproteobacteria*, or *Betaproteobacteria*. PERMANOVA analysis of SOX gene probes suggested a difference (*p* < 0.1) in response to As- and water treatments (Table 3). However, the PCA analysis of gene-probes for SOX genes showed a major separation between the rhizosphere compartments (Supplementary Figure 7). Major probes leading the divergence between the treatments represented a *Nitrobacter hamburgensis*, a *Rhodobacterales* sp., a *Rhodopseudomonas palustris*, a *Methylobacterium* sp., and a *Allochromatium vinosum.*

## Discussion

This study revealed that rice rhizosphere and root-plaque compartments are a niche for many distinct As-cycling functional groups and provided novel insights on their responses to water management and soil As-concentrations in a long term MSMA amended field site. Among several As-functional groups detected, ARBs, AMBs, and AOBs were major ones in both rhizosphere and root-plaque compartments. Although the relative abundance did not significantly change between the experimental variables (Figures 3, 4), the diversity of relative signal intensity based on PERMANOVA and PCA analysis revealed significant differences between the compartments and to some extent between the main treatments of arsenic and water management. Particularly, ARBs exhibited niche differentiation between the compartments and in response to long term As-contamination (Figure 3 and Supplementary Figure 4), probably enriching several ARBs resistant to higher As concentrations as noted in Supplementary Figures 4A,B. A previous study showed that several ARBs were enriched in As contaminated soils (Qin et al., 2006; Hu et al., 2019). One possible explanation was that *arsC* mediated reduction of As^V^ to As^III^ may also be a detoxification process for many microbes in As-enriched soils (Qin et al., 2006). Thus, it was presumed that bio-reduction of As^V^ to As^III^ by ARBs contributed to higher As^III^ concentrations in MSMA amended plots. Arsenic species quantification supported this assumption as As^III^ concentrations in the rhizosphere, root-plaque, pore water, and grain samples were significantly higher in CFA plots. Although, As^III^ was still detected in CFC plots and to a lesser extent in IFC plots, indicating the facultative nature of ARBs switching from anaerobic conditions and to less anaerobic conditions during the wet-dry cycles.

Relative abundance of several AMBs increased in both compartments under CF treatments, which accumulated significantly higher mAs concentrations in the grains, with highest concentrations detected in CFA plots. Moreover, mAs:total-As concentration ratios were consistently higher in the rhizosphere, root-plaque, pore water and grain samples within CF plots which clearly demonstrated higher methylation potential in both compartments under continuous flooding. Other studies have reported similar trends demonstrating that the more mAs was produced under continuous flooding compared to intermittently flooded practices (Li et al., 2009; Moreno-Jiménez et al., 2014). Higher As^III^ noted under CF plots may have subsequently triggered As-methylation in the rice rhizosphere, which is another detoxification process evolved by many microbes to detoxify more toxic As^III^ (Reid et al., 2017). One study noted that the higher As^III^ under continuous flooding practice enhanced As-methylating microbes compared to intermittently flooded rice (Zecchin et al., 2017b). Similarly, it was shown that higher concentrations of As^III^ stimulated As-methylation (Qin et al., 2006) and increased mAs concentrations in the rice grains (Wang et al., 2019). Thus, results of our study conform to the hypothesis that much of the grain mAs originates from the rhizosphere as a result of biotransformation by AMBs (Lomax et al., 2012). We further propose that root-plaque compartment is also a niche for microbial methylation of As and probably contributed to mAs uptake by rice plants. Intermittent flooding slightly decreased *arsM* abundance in the rhizosphere but did not completely inhibit their abundance as mAs was detected in rice grain from IF plots as well. Anaerobic As-methylators were probably diminished by intermittent water management (Reid et al., 2017), as predominant AMBs appeared to be anaerobic, and mostly within *Deltaproteobacteria*. However, it must be acknowledged that many diverse taxonomic groups have been identified with a capability to methylate As (Reid et al., 2017; Zhai et al., 2017; Afroz et al., 2019), which were not represented in GeoChip 4. Nonetheless, results of this study confirm the abundance of AMBs in the rice rhizosphere and their sensitivity to water management. Results further support the hypothesis that higher soil-As concentration increased ARB mediated As^III^ concentrations in the rhizosphere compartments, which potentially stimulated AMBs and generation of mAs in the rhizosphere and subsequent accumulation in the rice grain. Thus, demonstrating higher potential for microbial methylation of As in As contaminated rice fields under continuous flooding. This phenomenon might be responsible for higher mAs:AsIII concentrations in rice grains originating from long term MSMA contaminated plots, where intermittent water management led to a larger reduction in grain As^III^ and mAs-concentrations (Somenahally et al., 2011a, b).

Results also indicated that rice rhizosphere compartments are a niche for many pIRBs and SRBs, which distinctly assembled within the compartments and in response to water and As treatments. These predominantly anaerobic functional groups are supposedly sensitive to oxic-redox conditions but were only marginally diminished by more oxidized conditions at the root-plaque compartment. Transient anoxic conditions during root-respiration cycles probably induced their continued abundance and activity. Iron reduction and subsequent dissolution of complexed-As at the root-plaque compartment is critical as higher As:Fe ratios, particularly in CF plots, poses greater risk of releasing As bound to Fe-oxyhydroxides as noted in other studies (Jia et al., 2014; Hu et al., 2015). Additionally, some IRBs may have also contributed to reduction of As^V^ to As^III^, as many pIRBs were also positive for the *arsC* gene probes. Given the proximity to roots, these specialist pIRBs could release Fe-oxide bound As^V^ and also reduce it to As^III^, which is highly bioavailable and may also induce microbial methylation. Some released As may co-precipitate with sulfides, as noted by several studies (Newman et al., 1997; Cai et al., 2009; Battaglia-Brunet et al., 2012; Jia et al., 2015) or can also be incorporated into Fe^II^-sulfide minerals (Bostick and Fendorf, 2003). These processes can potentially minimize As-bioavailability, however, sulfide oxidizing bacteria could interrupt this mitigation potential by releasing sulfide bound-As through oxidation of thio-As compounds (Newman et al., 1997; Hoeft et al., 2004; Oremland et al., 2004). Although, it must be recognized that many microbial As-transformations and fate of bio-transformed-As compounds in the rice rhizosphere are still not clearly understood.

We further explored whether niche separation of ARBs, SRBs, and pIRBs within the rhizosphere compartments coupled to similar functional profiles. When other genomic functions were overlayed using CCA analysis, it was noted that ARBs in the root-plaque associated with nutrient cycling processes such as ammonification, phosphorus utilization and nitrification, whereas, in the rhizosphere ARBs were specialists for anaerobic metabolism of carbon through acetogenesis, methanogenesis and others. Differences in soil As-concentrations between these two compartments could be the primary driver of microbial assembly, as higher As concentrations generally promote functional groups that can also tolerate As-toxicity (Cai et al., 2009; Xiong et al., 2010). Moreover, CCA plots confirmed that As-resistance (*arsC*) was one of the major driving factors for SRBs in the root-plaque compartment. Association of As-transformation to different biogeochemical cycling processes was further evidenced in ARBs closely aligning with N-cycling processes, whereas As-methylation was linked to sulfate reduction in the rhizosphere. Thus, under long-term As contamination, continuous flooding may promote specialists linked to As-methylation within anaerobic functional groups such as SRBs. Thus, it can be proposed that wet-dry cycles under intermittent flooding elicited a major shift in AMBs and ARBs. For example, ARBs with assimilatory N-reduction and methane production were more sensitive to wet-dry cycles (intermittent flooding). Similar functional segregation was also observed for SRBs within the compartments, as SRBs in the rhizosphere associated with anaerobic N-cycling processes, whereas in the root-plaque with As-resistance mechanisms and nutrient utilization functions. It has been shown in other studies that As and S transformation processes are interlinked in SRBs, and may promote specialist functional groups in As-contaminated sites (Serrano and Leiva, 2017; Xue et al., 2020). Similarly, both water management and As-contamination have been noted to drive microbial specialist functional groups interlinked to As (Zecchin et al., 2017a). Similarly, another study noted that As-reduction was coupled to NO_3_ reduction in the flooded rice rhizosphere (Li et al., 2019), and it is probable for several of these N cycling microbes to gain As-transformation processes in As-contaminated soils. Thus, likelihood of microbial exposure to As and other nutrients appears to be a major factor driving the microbial assemblage toward specialists in As-resistance and nutrient cycling at the root-plaque. Other reports demonstrated similar mechanism of As-contamination influencing the metabolic coupling of As-transforming with Fe-cycling (Muehe et al., 2016; Yi et al., 2019), with S-cycling microorganisms (Das et al., 2021) and with metabolism of C, N, and P (Dunivin et al., 2019; Das et al., 2021).

## Conclusion

Results of this field study utilizing long-term MSMA contaminated rice field sites revealed niche-separation of functional microbiome in the rhizosphere compartments. Rhizosphere and root-plaque compartments assembled different ARBs and AMBs community, which were also responsive to water management and soil-As concentrations. As-transforming functional groups within the compartments coupled to different biogeochemical cycling processes confirming their distinctive functional capabilities for adapting to local biogeochemical conditions in the rhizosphere and the root-plaque compartments. These niche adaptations were also influenced by long-term As contamination, as higher potential for microbial As-reduction and As-methylation was noted in CFA plots. Intermittent flooding impacted these As-biotransformation processes and significantly reduced As^III^ and mAs accumulation in the grains. Study results provided a benchmark data on rhizosphere microbial biogeography and functional responses for predictive understanding of critical As-biotransformation processes in response to soil redox changes through water management and As contamination. These predictive responses could be modeled to develop mitigation strategies to alter key biotransformation processes such as As-reduction and As-methylation in soils with a history of As contamination.

## Data Availability Statement

The datasets presented in this study can be found in NCBI repository under the accession GSE179671.

## Author Contributions

AS, TG, and RL designed the study and experimental details. AS conducted the experiment, completed the analysis, and wrote the initial draft. TG, RL, and JZ contributed to the manuscript and editing. All authors contributed to the article and approved the submitted version.

## Conflict of Interest

The authors declare that the research was conducted in the absence of any commercial or financial relationships that could be construed as a potential conflict of interest.

## Publisher’s Note

All claims expressed in this article are solely those of the authors and do not necessarily represent those of their affiliated organizations, or those of the publisher, the editors and the reviewers. Any product that may be evaluated in this article, or claim that may be made by its manufacturer, is not guaranteed or endorsed by the publisher.
