# Supplementary material for: Niche Differentiation of Arsenic-Transforming Microbial Groups in the Rice Rhizosphere Compartments as Impacted by Water Management and Soil-Arsenic Concentrations
Source: Front Microbiol. 2021 Nov 5;12:736751. doi: 10.3389/fmicb.2021.736751 (PMC8602891; doi:10.3389/fmicb.2021.736751)
Supplement: Supplementary file 1 [file Data_Sheet_1.docx]

**Supplementary information**

**Materials and Methods**

**Rice cultivation and experimental treatments**

Rice cultivar ‘cocodrie’ (*Oryza japonica*) was used for this study. Approximately 1.5 g of dry seed was planted into a meter of drill. Four randomized replicated fields were established within MSMA and no-MSMA control blocks. The fields were further split to establish two water treatments. The nitrogen fertilizer (urea) was applied just before a permanent flood at five-leaf stage at 134 kg ha^−1^ of nitrogen.

**Rhizosphere and root-plaque sampling.**

Between 4-5 rice plants were collected from each experimental field plots, along with the adhering soil. The plants were vigorously shaken and then remaining loosely adhering non-rhizosphere soil and larger soil aggregates were manually removed. Plants were placed in ziplock bags to transport to the laboratory. After further inspection and scrubbing out potential non-rhizosphere soil leaving few millimeters around the roots, the rhizosphere soil was collected and stored appropriately. The roots were then thoroughly washed with sterile-deionized water to remove any remaining soil, with iron-oxide plaque remained firmly affixed to the roots. The root samples with plaque and the separate rhizosphere-soil samples, were each split into subsamples for subsequent chemical and microbial analyses.

**Pore-water sampling.**

Soil core samples from the rooting zone were collected using an 8.5 x 10-cm diameter core-auger with plastic liners. Soil samples collected within the plastic liners were capped with polypropylene end caps, which were further sealed to minimize aeration and stored on ice for transport to the laboratory. The core samples were vacuum filtered through 0.2µm pore size mixed cellulose-ester filters at a negative pressure of 138 kPa for 20 min to extract pore-water. The pore water collected was then acidified to pH 3 with HNO_3_ and stored for subsequent As analysis.

**Rice grain sampling**

Rice grain samples were obtained during harvest from each treatment plot. Entire plots were harvested and grain samples were separated. Approximately 50 g of representative grain samples from individual treatment plots were dehulled, milled, ground to flour and stored at 4 °C until further analysis.

**Redox potential measurement.**

Redox potential in the rhizosphere and bulk soil were measured in the field at 4 and 8 weeks after flooding and at the time of sampling using a platinum electrode connected to a silver (Ag) Ag/AgCl reference electrode. The platinum electrodes were inserted into the rooting zone and into the adjacent bulk soil (between two rows without roots). The electrodes were left undisturbed to equilibrate for 24 h before measuring the redox. While taking the redox measurement, the reference electrode was placed in the flood water. Further details on construction and platinum electrodes and procedures for measuring redox potential can be found in Fiedler et al. (2007) (1).

**Arsenic extraction and analysis from soil, root-plaque pore water and grain samples**

Sub samples stored in the freezer were thawed, and approximately 1 g of soil or 2.5 g of root-plaque samples were weighed to the third decimal and transferred into a 50 mL polypropylene centrifuge tubes. Ten mL of 0.4 M H_3_PO_4_ (pH 1.6) was added to the tubes, and the suspensions were agitated for 6 hr on a reciprocating platform shaker. The suspensions were then centrifuged at approximately 7500 x g for 5 min, and the supernatant was transferred to another tubes. Ten mL of M NaOH (pH~12.2) were added to the suspension residue, and were again agitated for 6 hr on a reciprocating platform shaker. Equal amounts from the two supernatant solutions were then diluted 100-fold using a solution of 2 mM HNO_3_ + 0.5 mM EDTA. The final sample matrix of 4 mM H_3_PO_4_ + 4 mM NaOH + 2 mM HNO_3_ + 0.5 mM EDTA at a pH ~3.0 was then analyzed for concentrations of various As-species.

The total-As concentrations in soil samples were extracted by following an open digestion method with HNO_3_/H_2_O_2_ as described in the EPA method-3050B (2). For rice grain samples, we used the trifluoroacetic acid (TFA) extraction method for extracting As species from grain samples following the method described in this study (3). Total grain-As concentration was determined following HNO_3_/H_2_O_2_ digestion as described in this study (4). Concentration of several As-species (AsV, AsIII, MMA, DMA) in the extracts obtained from rhizosphere, root-plaque and grain samples, and the pore-water samples were analyzed in a 200 model high performance liquid chromatography (HPLC) system (Perkin Elmer, Waltham, MA, USA) with a AG7 guard column (Dionex, Sunnyvale, CA, USA) and an IonPac AS7 anion-exchange column (Dionex), which were attached inline to a ELAN DRCII model inductively-coupled-plasma mass-spectrometry (ICP-MS) (Perkin Elmer). The total As concentrations were determined using a ELAN DRCII model ICP-MS (Perkin Elmer).

**Standards and reference materials used for As analysis**

The DMA^V^ and MMAs^V^ were obtained from Chem Service (West Chester, PA, USA) as dimethyl arsinic acid and monosodium acid methanearsonate, respectively. Arsenate was obtained as sodium arsonate (Na_2_HAsO_4_•7H_2_O) from Sigma (St. Louis, MO, USA) and iAs^III^ as arsenite oxide (As_2_O_3_) from Alfa Aesar (Ward Hill, MA, USA). A Perkin Elmer 200 HPLC system (Waltham, MA, USA) with a guard column (Dionex IonPac AG7, Sunnyvale, CA, USA) and an anion-exchange column (Dionex IonPac AS7) was used for separation of As species, which were then quantified by in-line ICP-MS using a Perkin Elmer DRC-ELAN II. The HPLC instrument parameters are presented in Table S3. The post column addition of 3% methanol was used to offset ionization problems due to variable C concentrations. The total As was measured by ICP-MS.

**Estimation of Fe concentrations in root-plaque samples:**

In order to estimate As:Fe molar ratios in the root-plaque samples, a separate total-As and Fe concentrations were determined following ammonium-oxalate extraction in the dark . Approximately 2.5 g of fresh root was placed in a polypropylene centrifuge tube that was covered with aluminum foil to prevent exposure to light. Twenty milliliters of extracting solution (0.175 M ammonium oxalate + 0.1 M oxalic acid at pH 3) were added and the resulting mixture was agitated for 2 hr on a reciprocating platform shaker. The suspensions were then centrifuged, and the supernatant solution was filtered, and stored at 4 ^o^C for As and Fe analysis. The dry mass of the root-plaque samples were analyzed after the samples were washed with deionized water and oven dried at 60 ^o^C for 12 hours. Total iron concentrations were determined using an AAnalyst 400 model atomic-absorption spectrophotometer (Perkin Elmer).

**GeoChip microarray hybridization**

The genomic DNA samples extcrated from the rhizosphere and root-plaque samples were labelled with Cy-3, purified using the QIA quick purification kit (Qiagen, Valencia, CA, USA) and quantified in a NanoDrop ND-1000 spectrophotometer (ThermoSavant, Milford, MA, USA) and mixed with sample tracking control (NimbleGen). Hybridization buffer at 7.32 μL/sample was added, which contained 40% formamide, 1% SDS, 2.38% Cy3-labelled alignment oligo (NimbleGen), 25% SSC and 2.8% Cy5-labelled CORS target. The samples were then mixed by vortexing, centrifuged, incubated at 95 °C for 5 min and maintained at 42 °C until hybridization. An HX12 mixer (NimbleGen) was placed onto the array using NimbleGen's precision mixer alignment tool, and then, the array was preheated to 42 °C on a hybridization station (MAUI, BioMicro Systems, Salt Lake City, UT, USA) for 5 min. Samples (6.8 μL) were then loaded onto the array surface and hybridized approximately 16 h with mixing.

After hybridization, arrays were scanned at 100% photomultiplier tubes gain using a NimbleGen MS 200 Microarray Scanner (Roche NimbleGen). Scanned images were assigned grids using the NimbleScan software. Signal intensity for each probe were obtained using the grid file containing GeoChip 4 probes and NimbleGen control probes. Probe spots with coefficient of variance (CV) >0.8 were removed. Furter details on image processing details can be found in Tu et al. (2014) ((5)

**Supplementary tables and figures**

Table S1. Chemical characteristics of soil samples from the treatment plots

| Treatments | Soil Texture | Soil pH | EC  (µmhos cm^-1^) | Organic carbon | Total N |  | NO_3_ | P | K | Ca | Mg | S | Na | Fe | Zn |
| --- | --- | --- | --- | --- | --- | --- | --- | --- | --- | --- | --- | --- | --- | --- | --- |
|  |  |  |  | % | |  | mg kg^-1^ | | | | | | | | |
| CFA | Silt loam | 5.78 | 141 | 0.81 | 0.11 |  | 8.6 | 20.5 | 148 | 800 | 124 | 18.5 | 189 | 55.2 | 2.8 |
| CFC | Silt loam | 5.89 | 189 | 0.89 | 0.12 |  | 9.4 | 28.4 | 158 | 849 | 138 | 15.9 | 135 | 48.5 | 3.1 |
| IFC | Silt loam | 5.94 | 194 | 0.87 | 0.13 |  | 8.9 | 31.4 | 178 | 950 | 189 | 16.8 | 141 | 42.8 | 5.9 |

Table S2. Geochip gene probes used in this study for different functional groups

| Category | Gene probes |
| --- | --- |
| Arsenic | aoxB, arsA/B/C, arsM |
| Acetogenesis | FTHFS |
| Adenylylsulfate reductase | APS_AprA/B/ AprA |
| Ammonification | ureC |
| Aromatics remediation | 4HBH, akbF, BADH, bclA, bco, benAB, bphA/C/F1, carA, catB, cumA, GCoADH, HBH, hmgA/C, mdlA/B/C, mhpA, nagG/'/k, nahA, nhh, nitA, nmoA, ohbAB, pcaG, PhaB, pheA, pimF, pmdAB, PobA, xylC/G |
| Assimilatory N reduction | nasA, NiR, NiRA/B |
| Cadmium | CadA, czcA |
| Carbon degradation | AceA/b, acetylglucosaminidase, amyA/X, ara, cda, CDH, cellobiase, endochitinase, endoglucanase, exochitinase, exoglucanase, glucoamylase, glx, limEH, LMO, mannanase, nplT, pectinase, phenol_oxidase, pulA, vanA, vdh, xylA, xylanase |
| Carbon fixation | aclB, CODH, pcc, RUBISCO |
| Cytochrome | cytochrome_b/c |
| Denitrification | narG, nirK, nirS, nosZ |
| Dissimilatory N reduction | napA, nrfA |
| Methane | mcrA, mmoX |
| Phosphate limitation | phoA/B, pstA/B/C |
| Phosphorus utilization | ppk, ppx |
| Sulfite reductase | dsrA/B |
| Sulphur oxidation | sox |


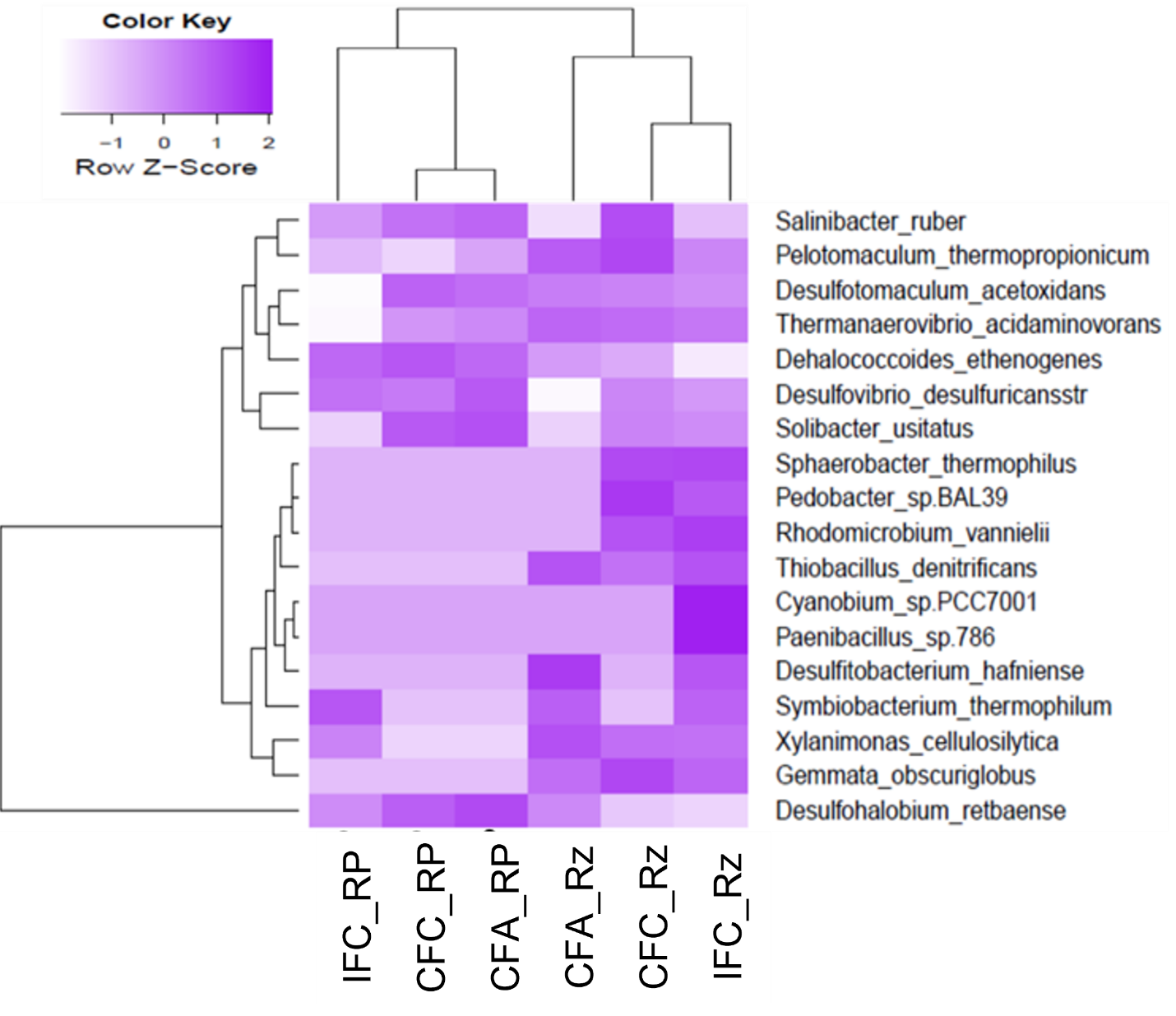


Figure S1. Hierarchical cluster analysis of relative abundance of *arsM* gene containing prokaryotes detected in the rhizosphere compartments under different soil-As concentrations and water management treatments. Higher intensity of color corresponds to higher abundance. CFA=continuously flooded-arsenic, CFC=continuously flooded-control and IFC = intermittently flooded control. Suffix RP and Rz represent the root-plaque and the rhizosphere samples, respectively.


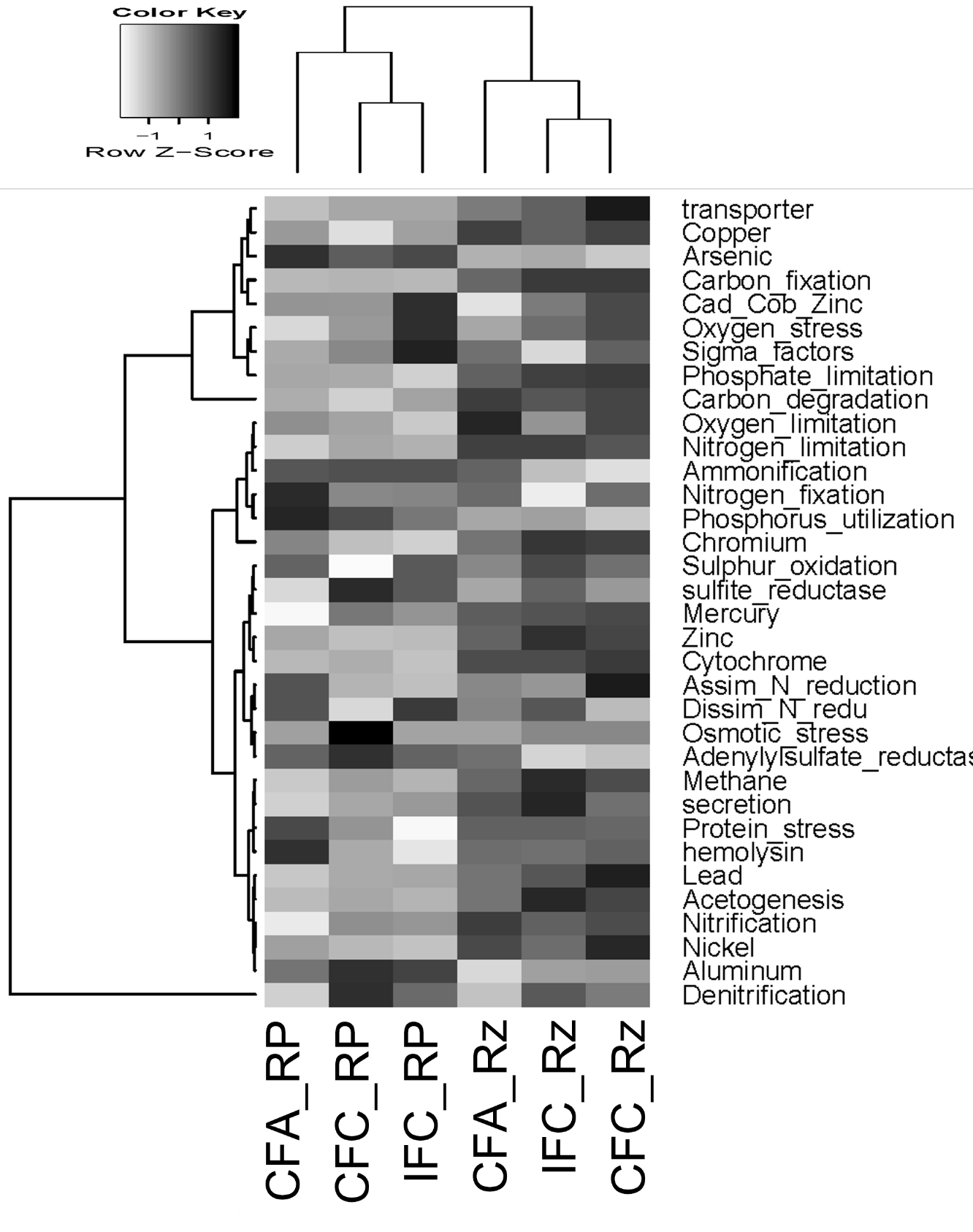


Figure S2. Cluster analysis for other functional gene probes detected for ARBs. This analysis was performed in addition to CCA analysis to reveal the differences between the treatments Higher intensity of color corresponds to higher abundance. CFA=continuously flooded-arsenic, CFC=continuously flooded-control and IFC = intermittently flooded control. Suffix RP and Rz represent the root-plaque and the rhizosphere samples, respectively.


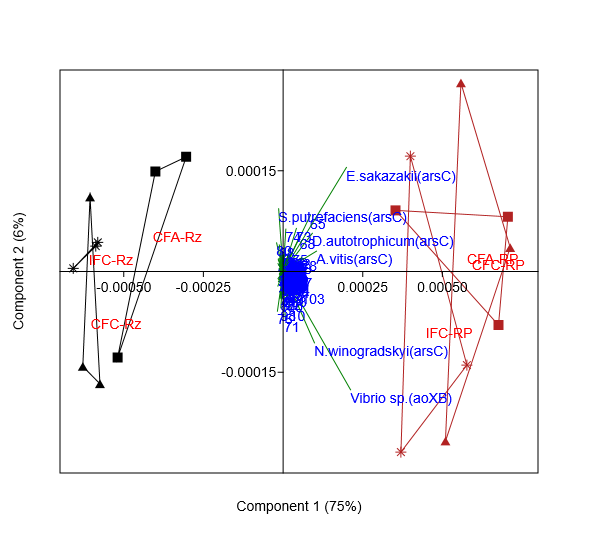


Figure S3. PCA ordination for all arsenic transforming gene-probes detected in Geochip. The values in parenthesis next to components 1 and 2 represent the percentages of variance accounted in each component. Axis variables (gene probe OTU IDs) are projected as bi-plot vectors, and some major vectors are labeled to reveal the species name. Suffix RP and Rz in group labels represent the root-plaque and the rhizosphere samples, respectively. CFA=continuously flooded-arsenic (squares), CFC=continuously flooded-control (triangles) and IFC = intermittently flooded control (stars).


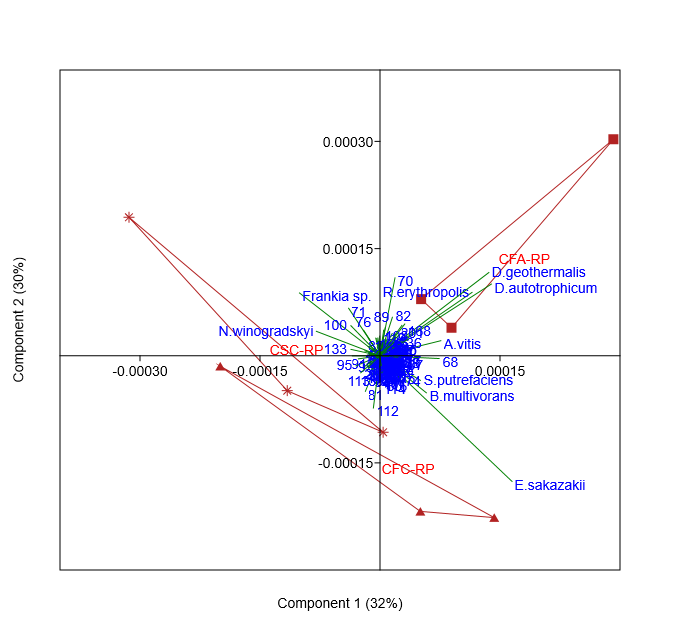

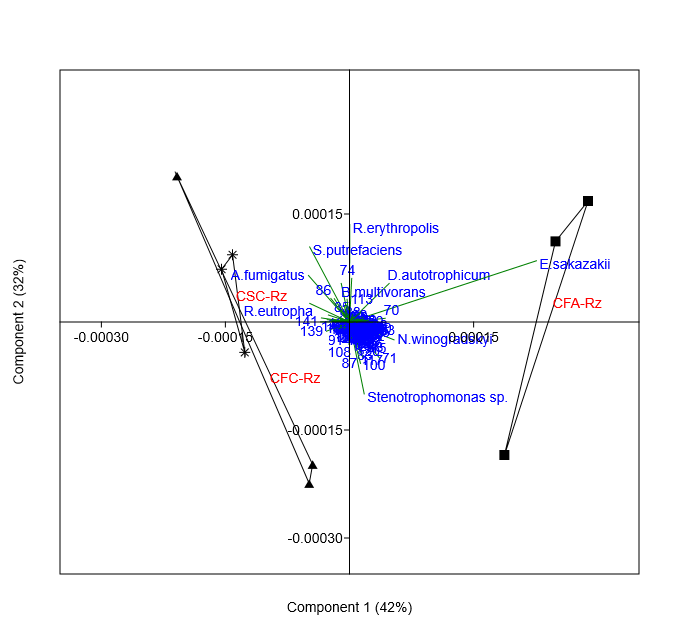


Figure S4. PCA ordination for ARBs in the rhizosphere (a) and the root-plaque (b) compartments. The values in parenthesis next to components 1 and 2 represent the percentages of variance accounted in each component. Axis variables (gene probe OTU IDs) are projected as bi-plot vectors, and some major vectors are labeled to reveal the species name. Suffix RP and Rz in group labels represent the root-plaque and the rhizosphere samples, respectively. CFA=continuously flooded-arsenic (squares), CFC=continuously flooded-control (triangles) and IFC = intermittently flooded control (stars).


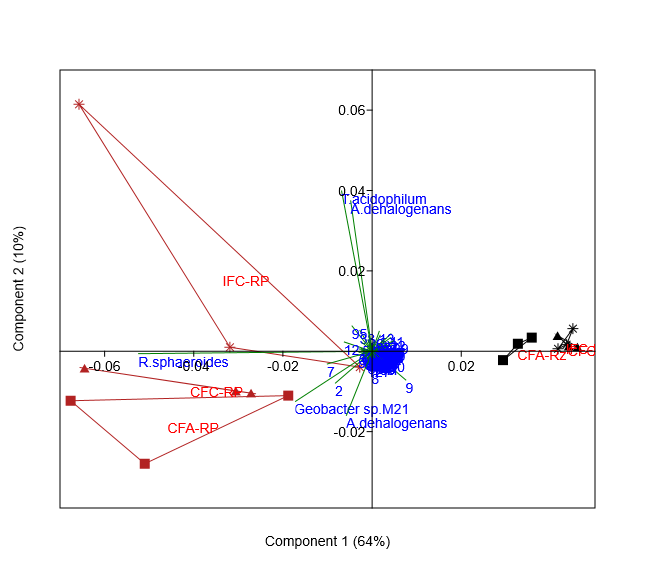


Figure S5. PCA ordination for potential IRBs detected in Geochip. The values in parenthesis next to components 1 and 2 represent the percentages of variance accounted in each component. Axis variables (gene probe OTU IDs) are projected as bi-plot vectors, and some major vectors are labeled to reveal the species name. Suffix RP and Rz in group labels represent the root-plaque and the rhizosphere samples, respectively. CFA=continuously flooded-arsenic (squares), CFC=continuously flooded-control (triangles) and IFC = intermittently flooded control (stars).


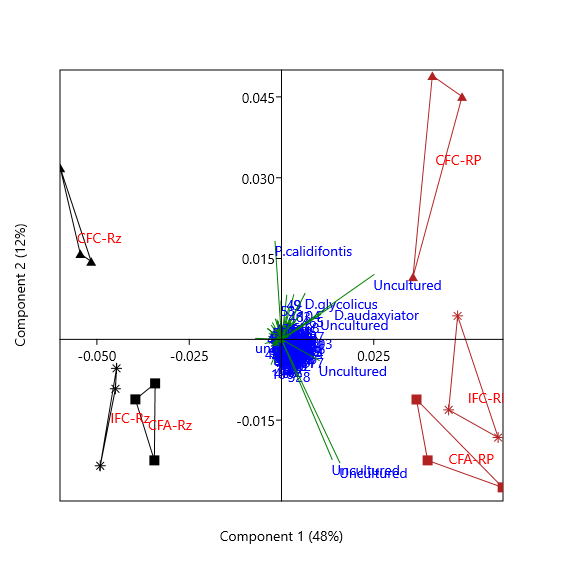


Figure S6. PCA ordination for SRBs (dsrA/B, APS_AprA/B gene probes) detected in Geochip. The values in parenthesis next to components 1 and 2 represent the percentages of variance accounted in each component. Axis variables (gene probe OTU IDs) are projected as bi-plot vectors, and some major vectors are labeled to reveal the species name. Suffix RP and Rz in group labels represent the root-plaque and the rhizosphere samples, respectively. CFA=continuously flooded-arsenic (squares), CFC=continuously flooded-control (triangles) and IFC = intermittently flooded control (stars).


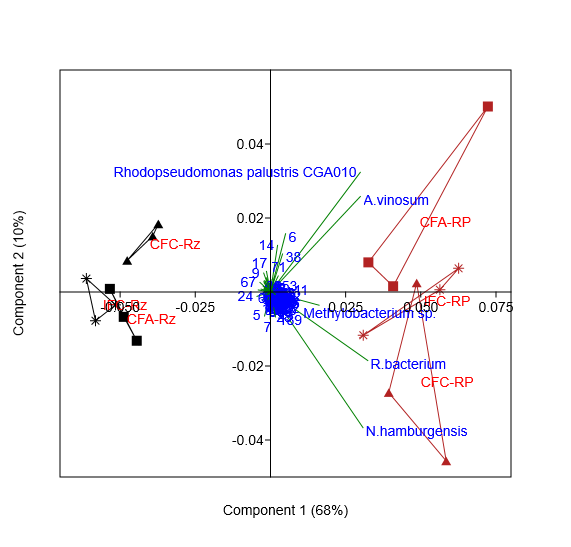


Figure S7. PCA ordination for SOBs (SOX gene probes) detected in Geochip. The values in parenthesis next to components 1 and 2 represent the percentages of variance accounted in each component. Axis variables (gene probe OTU IDs) are projected as bi-plot vectors, and some major vectors are labeled to reveal the species name. Suffix RP and Rz in group labels represent the root-plaque and the rhizosphere samples, respectively. CFA=continuously flooded-arsenic (squares), CFC=continuously flooded-control (triangles) and IFC = intermittently flooded control (stars).

**References**

1. Fiedler, S., Vepraskas, M.J., Richardson, J. (2007). Soil redox potential: importance, field measurements, and observations. Advances in agronomy. 94, 1-54.

2. USEPA, E. (1996). Method 3050B-Acid digestion of sediments, sludges and soils. Test methods for evaluating solid wastes: physical/chemical methods. EPA SW‐846. 1, 3050B-1.

3. Heitkemper, D.T., Vela, N.P., Stewart, K.R., Westphal, C.S. (2001). Determination of total and speciated arsenic in rice by ion chromatography and inductively coupled plasma mass spectrometry. Journal of Analytical Atomic Spectrometry. 16(4), 299-306.

4. Pillai, T.R., Yan, W., Agrama, H.A., James, W.D., Ibrahim, A.M.H., McClung, A.M., et al. (2010). Total Grain-Arsenic and Arsenic-Species Concentrations in Diverse Rice Cultivars under Flooded Conditions. Crop Sci. 50(5), 2065-2075.

5. Tu, Q., Yu, H., He, Z., Deng, Y., Wu, L., Van Nostrand, J.D., et al. (2014). GeoChip 4: a functional gene‐array‐based high‐throughput environmental technology for microbial community analysis. Mol. Ecol. Resour. 14(5), 914-928.
